# Supplementary material for: End Sequence Analysis Toolkit (ESAT) expands the extractable information from single-cell RNA-seq data
Source: Genome Res. 2016 Oct;26(10):1397–410. doi: 10.1101/gr.207902.116 (PMC5052061; doi:10.1101/gr.207902.116)
Supplement: Supplemental Material [file supp_gr.207902.116_Supp_Materials.tar.gz › ESAT-esat_v0.1/doc/html/classbroad_1_1pda_1_1seq_1_1rap_1_1_r_a_p_peak_caller.html]

ESAT: broad.pda.seq.rap.RAPPeakCaller Class Reference


ESAT


- Main Page
- Related Pages
- Packages
- Classes
- Files

- Class List
- Class Index
- Class Hierarchy
- Class Members

All Classes Namespaces Files Functions Variables Enumerator Pages

- broad
- pda
- seq
- rap
- RAPPeakCaller

Public Member Functions |
Static Public Member Functions |
Static Protected Attributes |
List of all members

broad.pda.seq.rap.RAPPeakCaller Class Reference

Collaboration diagram for broad.pda.seq.rap.RAPPeakCaller:

[legend]

|  |  |
| --- | --- |
| Public Member Functions | |
|  | RAPPeakCaller (String backgroundFile, String rapFile, String sizes, String maskedregions, int windowSize, int step) throws IOException |
|  | |
| void | callSkellamPeaks (String outFile, double maxSkellam, double maxScanPvalue) throws IOException |
|  | |
| void | callKLPeaks (String outFile, double klCutOff) throws IOException |
|  | |
| CoordinateSpace | getCoordinateSpace () |
|  | |

|  |  |
| --- | --- |
| Static Public Member Functions | |
| static void | main (String[] args) throws IOException |
|  | |

|  |  |
| --- | --- |
| Static Protected Attributes | |
| static Logger | logger = Logger.getLogger(RAPPeakCaller.class.getName()) |
|  | |

## Detailed Description

Author
:   skadri

## Constructor & Destructor Documentation

|  |  |  |  |
| --- | --- | --- | --- |
| broad.pda.seq.rap.RAPPeakCaller.RAPPeakCaller | ( | String | *backgroundFile*, |
|  |  | String | *rapFile*, |
|  |  | String | *sizes*, |
|  |  | String | *maskedregions*, |
|  |  | int | *windowSize*, |
|  |  | int | *step* |
|  | ) |  | throws IOException |

Parameters
:   |  |  |
    | --- | --- |
    | backgroundFile | Alignment file for the input sample |
    | rapFile | Alignment file for the rap file |
    | sizes | Chromosome sizes |
    | windowSize | Window Size |
    | step | Step Size / Overlap between consecutive windows |
    | maxSkellam |  |
    | maxScanPvalue |  |

Exceptions
:   |  |  |
    | --- | --- |
    | IOException |  |

Here is the caller graph for this function:

## Member Function Documentation

|  |  |  |  |
| --- | --- | --- | --- |
| void broad.pda.seq.rap.RAPPeakCaller.callKLPeaks | ( | String | *outFile*, |
|  |  | double | *klCutOff* |
|  | ) |  | throws IOException |

For each window, calculate the poisson lambda for the given window over the entire chromosome

Parameters
:   |  |  |
    | --- | --- |
    | model |  |
    | window |  |
    | windowSize |  |

Returns

Exceptions
:   |  |  |
    | --- | --- |
    | IOException |  |

|  |  |  |  |
| --- | --- | --- | --- |
| void broad.pda.seq.rap.RAPPeakCaller.callSkellamPeaks | ( | String | *outFile*, |
|  |  | double | *maxSkellam*, |
|  |  | double | *maxScanPvalue* |
|  | ) |  | throws IOException |

Call peaks using skellam

Parameters
:   |  |  |
    | --- | --- |
    | outFile |  |

Exceptions
:   |  |  |
    | --- | --- |
    | IOException |  |

|  |  |  |  |  |
| --- | --- | --- | --- | --- |
| CoordinateSpace broad.pda.seq.rap.RAPPeakCaller.getCoordinateSpace | ( |  | ) |  |

Returns the coordinate space for this peak caller

|  |  |  |  |  |  |
| --- | --- | --- | --- | --- | --- |
| static void broad.pda.seq.rap.RAPPeakCaller.main | ( | String[] | *args* | ) | throws IOException |

static

Parameters
:   |  |  |
    | --- | --- |
    | args |  |

Exceptions
:   |  |  |
    | --- | --- |
    | IOException |  |

Here is the call graph for this function:

## Member Data Documentation

Logger broad.pda.seq.rap.RAPPeakCaller.logger = Logger.getLogger(RAPPeakCaller.class.getName())

staticprotected

---

The documentation for this class was generated from the following file:

- java/broad/pda/seq/rap/RAPPeakCaller.java


---

Generated on Fri Aug 15 2014 15:11:03 for ESAT by  

 1.8.7
